# Investigation of Antihypertensive Properties of Chios Mastic via Monitoring microRNA-21 Expression Levels in the Plasma of Well-Controlled Hypertensive Patients

**DOI:** 10.3390/ncrna10030033

**Published:** 2024-05-31

**Authors:** Maria Tsota, Panagiota Giardoglou, Evangelia Mentsiou-Nikolaou, Panagiotis Symianakis, Ioanna Panagiota Kalafati, Anastasia-Areti Kyriazopoulou-Korovesi, Lasthenis Angelidakis, Maria Papaioannou, Christina Konstantaki, HYPER-MASTIC Consortium, Kimon Stamatelopoulos, George V. Dedoussis

**Affiliations:** 1 Department of Nutrition-Dietetics, School of Health Science and Education, Harokopio University, 17676 Athens, Greece; mtsota75@gmail.com (M.T.); evemen@hua.gr (E.M.-N.);; 2 Department of Clinical Therapeutics, School of Medicine, National and Kapodistrian University of Athens, 11528 Athens, Greece; anastasiakyko@gmail.com (A.-A.K.-K.); stamatelopoulosk@yahoo.gr (K.S.); 3 Department of Pharmacy, National and Kapodistrian University of Athens, 15771 Athens, Greece; 4 Department of Biology, National and Kapodistrian University of Athens, 15772 Athens, Greece

**Keywords:** hypertension, Chios mastic, biomarker, epigenetics, microRNA-21

## Abstract

Hypertension is a chronic, multifactorial disease, leading to high cardiovascular morbidity and mortality globally. Despite the advantages of pharmaceutical treatments, natural products have gained scientific interest due to their emerging phytotherapeutic properties. Chios mastic is a natural Greek product, consisting of bioactive compounds which modify microRNAs’ (small, expression-regulating molecules) expression. In this study, we investigated the antihypertensive properties of Chios mastic through the assessment of miR-21 levels. Herein, plasma samples of 57 individuals with hypertension, recruited for the purposes of the HYPER-MASTIC study, were analyzed. This was a clinical trial with Chios mastic supplements in which the patients were divided into groups receiving high and low mastic doses and placebo supplements, respectively. miR-21 was significantly upregulated in patients compared to normotensive individuals. Mean changes in miR-21 levels were statistically significant, after adjusting for sex and age, between the placebo and low-dose group and between the low- and high-dose group. Post-intervention miR-21 levels were positively associated with night-time systolic blood pressure, pulse pressure, and central systolic mean arterial pressure and negatively associated with night-time pulse wave velocity in the low-dose group. Our findings suggest a potential implication of miR-21 in the association of Chios mastic with night-time blood pressure measurements.

## 1. Introduction

Hypertension is a multifactorial, chronic, non-communicable disease, which is clinically described by a systolic blood pressure (SBP) ≥ 140 mmHg and a diastolic blood pressure (DBP) ≥ 90 mmHg [1]. It is partly attributed to genetic and epigenetic factors [2], as well as other non-modifiable (i.e., sex and nationality) and modifiable factors (i.e., lifestyle patterns and dietary habits) [3,4]. It constitutes a public health burden and a major cause of morbidity worldwide due to its implication in cardiovascular and chronic kidney diseases [5,6]. Although the majority of current therapeutic strategies seem to have a significant effect, hypertension remains a life-threatening disease. Among others, treatment of patients with hypertension usually involves a combination of drugs, leading not only to poor adherence but also to high-cost therapy [1].

Hypertension pathophysiology is versatile and includes diverse causal mechanisms such as smooth muscle cell differentiation [7,8], endothelial dysfunction [9,10], angiogenesis [11,12], inflammation [13], oxidative stress [14], and renin–angiotensin–aldosterone system (RAAS)-mediated sympathetic nervous system activity [15]. Thus, its complex etiology and disease progression pathways need deeper clarification in order to sub-dissect the underlying mechanisms of the disease.

Due to the significant impact of lifestyle factors on the onset and progress of hypertension, epigenetic mechanisms play a pivotal role in the regulation of genes involved in all the aforementioned pathophysiological conditions. MicroRNAs (miRNAs) are small (approximate length of 22 nucleotides), endogenous, single-stranded, non-coding RNA molecules, which exert post-transcriptional regulation of gene expression [6,16,17,18]. miRNAs control gene expression via binding on the 3′ untranslated region (3′UTR) of target-mRNAs, leading to mRNA degradation and/or translation obstruction and, therefore, inhibition of protein formation [19,20]. Due to the extreme stability of circulating microRNAs in blood and their responsive expression levels according to the progression of several pathological conditions, they have arisen as powerful biomarkers for the prognosis and monitoring of chronic diseases [21].

Specifically, microRNA-21 has emerged as a hypertension biomarker supported by a plethora of studies since its expression levels are upregulated in the tissues of individuals with hypertension, compared to healthy controls [22,23,24,25,26]. The unraveling of several miR-21 target-genes has corroborated its implication in signaling pathways, related to crucial hypertension mechanisms. Evidence suggests that miR-21 levels correlate with smooth muscle cell differentiation, proliferation, and contraction [27,28,29,30]. In addition, several studies have shown engagement of miR-21 with diverse pathophysiological pathways, such as endothelial dysfunction and angiogenesis inhibition [31,32], oxidative stress [22,32,33], RAAS signaling [34], cardiac fibrosis [35,36,37], and immunosuppression [38].

Several research groups have attempted to decipher the interplay between nutrients, distinct bioactive compounds, and microRNA expression levels in numerous cell types and tissues, hence expanding the existing knowledge in the newly formed field of nutrimiromics [21,39]. Chios mastic is a natural product, isolated from the evergreen shrub of *Pistacia lentiscus* L. var. *Chia*, which is exclusively cultured and harvested in the South part of the Greek island of Chios [40]. Even though it has been used since antiquity for medicinal and culinary purposes [41], it is only during the last two decades that researchers have focused on the investigation of its antihypertensive properties [42]. Specifically, several studies have verified Chios mastic’s antiatherogenic [43,44,45], hypolipidemic [46,47,48], anti-inflammatory [49,50,51,52], and antioxidant [53,54] effects.

In this study, we aimed to evaluate the antihypertensive effect of Chios mastic supplementation, adjunct to the appropriate medication, in a sample of well-controlled patients with hypertension, based on the expression levels of miR-21. For this purpose, a randomized, double-blind, placebo-controlled clinical trial was designed, in which individuals with hypertension consumed, on a daily basis, either placebo or Chios mastic supplement in two different concentrations. Initially, we analyzed the miR-21 expression levels of patients with hypertension compared to normotensive individuals, and following this, we examined the potential fluctuation of the plasma miR-21 expression levels of each intervention group caused by Chios mastic and the possible association of miR-21 with hypertension indices. 

## 2. Results

### 2.1. miR-21 Expression Is Upregulated in Individuals with Hypertension

**Figure 1:**
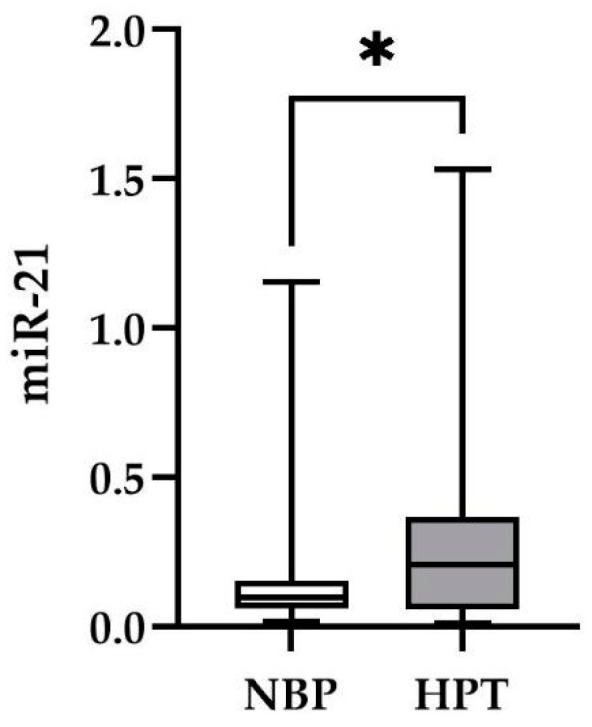
miR-21 levels in the plasma of individuals with normal blood pressure and hypertension. NBP: normal blood pressure; HPT: hypertension. Data are represented as mean ± SEM. * significantly different *p*-value ≤ 0.05.

As a proof of concept, in order to confirm the miR-21 utilization as a hypertension biomarker, we calculated its expression levels in two distinct groups: individuals with hypertension and individuals with normal blood pressure (normotensive controls). To do so, qRT-PCR was performed in plasma samples of 57 hypertensive patients and 46 normotensive individuals. In accordance with previous findings, miR-21 levels were significantly higher in individuals with hypertension when compared to those with normal blood pressure (*p* = 0.010) (Figure 1). Thus, we demonstrated the feasibility of miR-21 as a robust biomarker for hypertension and its reliability as a molecular tool for the analyses conducted during the current study.

### 2.2. Study Population Characteristics

Samples derived from 57 individuals with hypertension were used for the current analysis. Volunteers were randomly allocated into three intervention groups: 23 patients (40%) were allocated in the placebo group, 16 (28%) in the low-dose mastic group, and 18 (32%) in the high-dose mastic group. Baseline anthropometric measurements, medical records, and miR-21 levels for each intervention group are presented in Table 1. No statistically significant differences were observed between the three groups. Moreover, baseline measurements of hemodynamic and vascular function parameters for the three intervention groups are also presented in Table 1. Likewise, we did not observe any statistically significant differences between the groups.

After comparing the anthropometric, clinical (Supplementary Materials), hemodynamic, and vascular parameters, we did not detect any statistically significant differences among the three intervention groups. These analyses were conducted in order to ensure the homogeneity of our sample and attribute any observed difference in miR-21 levels to the additive effect of Chios mastic.

### 2.3. Monitoring of Plasma miR-21 Expression Levels in Patients with Hypertension

The main goal of the current study was to detect potential differences of hypertensive properties in patients with hypertension after the consumption of Chios mastic supplement. Therefore, we first investigated prospective changes in miR-21 expression levels between the baseline and upon completion of intervention for each group (*p*_time_), and following this, we examined potential differences in the mean changes in miR-21 levels between the different groups (*p*_unadj_ and *p*_adj_ for each comparison). qRT-PCR was conducted in order to determine the relative quantification of miR-21 levels in the plasma of patients with hypertension at the beginning and the end of the three-month intervention, adjunct to anti-hypertensive treatment. 

Expression levels of miR-21 in the plasma, at baseline, and post-intervention are presented for each group in Table 2. None of the intervention groups showed any statistically significant change in miR-21 plasma levels after the intervention (Table 2, *p*_time_). 

Subsequently, we compared mean differences of miR-21 expression levels (baseline to post-intervention) among intervention groups. We did not observe statistically significant differences for any of these comparisons (Table 2). Following, in order to correct for improprieties and to eliminate confounding factors, we performed statistical adjustment for sex and age (*p*_adj1_), as well as for sex, age, and BMI categories (*p*_adj2_). BMI categories were formed as follows: category 1 when BMI ≤ 24.9 kg/m^2^, category 2 when 25 kg/m^2^ ≤ BMI ≤ 29.9 kg/m^2^, category 3 when 30 kg/m^2^ ≤ BMI ≤ 34.9 kg/m^2^, category 4 when 35 kg/m^2^ ≤ BMI ≤ 39.9 kg/m^2^, and category 5 when BMI ≥ 40 kg/m^2^. We observed a statistically significant difference in mean change of miR-21 levels between the placebo and the low-dose mastic group (*p*_adj1_ = 0.037) and between the low- and high-dose mastic group (*p*_adj1_ = 0.018) when adjusting for age and sex. In addition, when adjusting for age, sex, and BMI categories, we observed significant differences between the aforementioned groups (*p*_adj2_ = 0.017 and *p*_adj2_ = 0.010, respectively).

### 2.4. miR-21 Levels Are Associated with Hemodynamic and Vascular Function Parameters in the Low-Dose Mastic Group Post-Intervention

**Figure 2:**
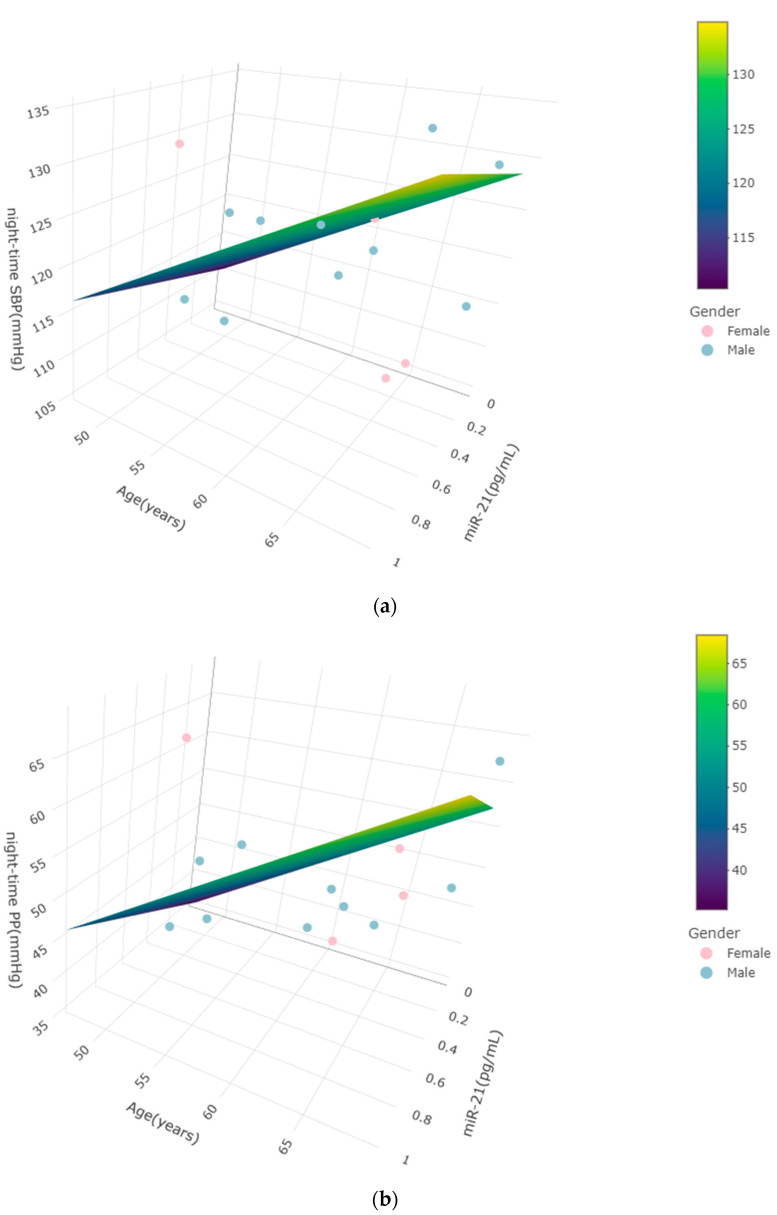

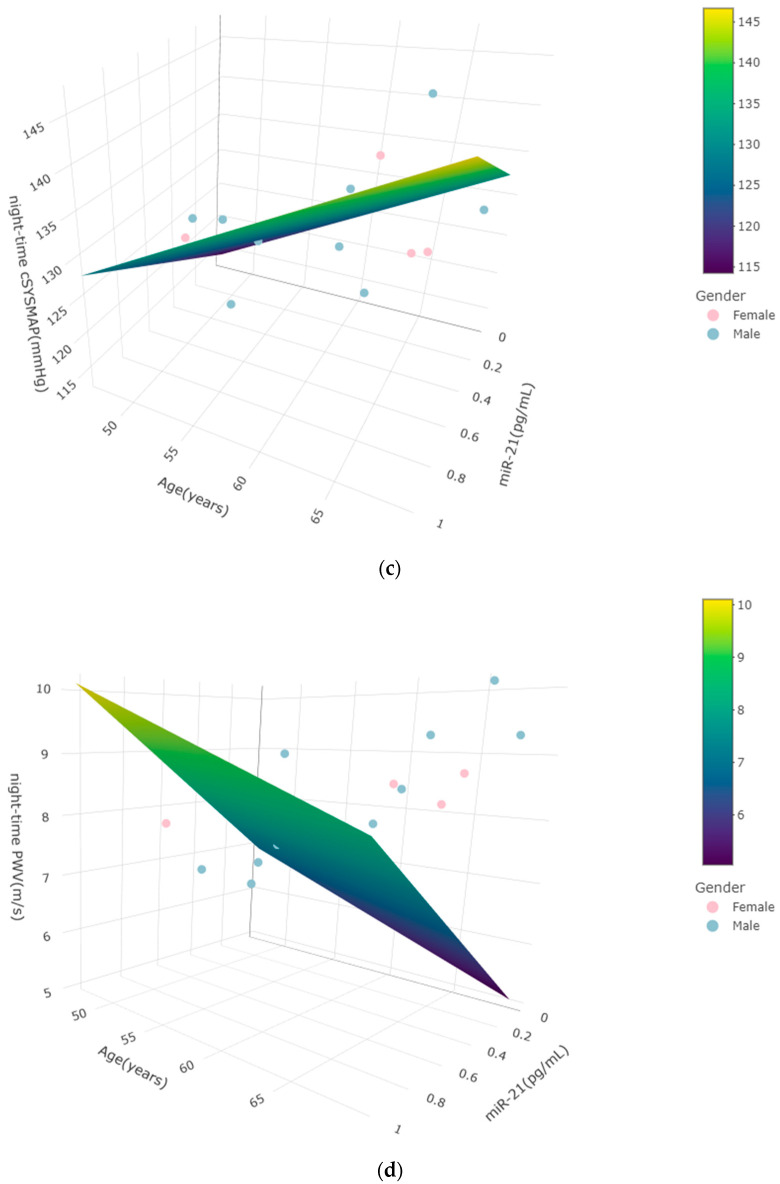
Multilinear regression analysis of miR-21 levels visualizing night-time blood pressure parameters, post-intervention, in the low-dose of mastic group. Panel axes represent miR-21 levels, age, and a response variable. (**a**) Night-time systolic blood pressure; (**b**) night-time pulse pressure; (**c**) night-time central systolic mean arterial pressure; (**d**) night-time pulse wave velocity. The color of the plane is depicted according to the variable’s value. Pink and blue dots represent female and male individuals, accordingly. Figures were designed using the “Plotly” package from R.

Linear and multilinear regression models (the latter adjusted for sex and age) were used to examine possible correlations between miR-21 levels, pre- and post-intervention, with anthropometric (Supplementary Materials), clinical (Supplementary Materials), hemodynamic, and vascular parameters (Supplementary Materials). At baseline, multilinear regressions were performed for all of the patients, and no significant association was observed for the aforementioned parameters. Upon completion of the three-month intervention, multilinear regression was carried out, examining association of the miR-21 levels with the same parameters for each intervention group. Analysis showed a significant association of miR-21 expression with several night-time measurements in the low-dose mastic group (Supplementary Materials). Specifically, miR-21 levels were positively associated with night-time SBP (β = 17.870; *p* = 0.032) (Figure 2a), PP (β = 20.968; *p* = 0.006) (Figure 2b), and cSMAP (β = 17.706; *p* = 0.023) (Figure 2c) and negatively with PWV (β = −1.783; *p* < 0.001) (Figure 2d). Our findings suggest a possible effect of Chios mastic on night-time blood pressure parameters through the regulation of miR-21 expression. Specifically, the positive associations with night-time hypertension indices, such as SBP, PP, and cSMAP, might indicate a Chios-mastic-mediated induction of miR-21 activity. Also, the negative association of miR-21 levels with PWV might imply a regulatory effect of Chios mastic on miR-21 implication in arterial stiffening pathways. No significant associations were observed for 24 h and daytime measurements.

## 3. Discussion

In this study, we sought to investigate the anti-hypertensive effect of Chios mastic supplementation through miR-21 expression assessment in an attempt to provide a new perspective on epigenetic regulators of hypertension mechanisms. 

microRNAs are ubiquitous, stable RNA molecules which play a crucial role in various pathophysiological pathways as expression regulators and herald great potential as disease biomarkers. Due to its regulatory function in hypertensive pathways, miR-21 constitutes a strong biomarker for hypertension monitoring. Many studies have already confirmed the elevated expression levels of miR-21 in hypertensive individuals via the comparison to those of normotensive ones [22,23,24,25,26]. This positive correlation has also been verified in our study population when comparing miR-21 plasma levels of hypertensive volunteers to non-hypertensive individuals.

In particular, miR-21 shows a positive association with left ventricular mass index, an established result of persistently high blood pressure [25] that has a regulatory effect on genes essential for vascular smooth muscle cell contraction, proliferation, and migration [28,29,30]. Furthermore, it is known that miR-21 represses RhoB expression in endothelial cells, leading to angiogenesis inhibition [31], and regulates superoxide dismutase 2 (SOD2), as well as Sprouty 2, resulting in NO (nitric oxide) reduction and endothelial dysfunction [32]. In a hypertensive rat model, cytochrome b, a protein encoded by mitochondrial DNA, has been identified as a direct target of miR-21. Its negative regulation results in elevated ROS (reactive oxygen species) production and oxidative stress exacerbation [33]. The versatile effect of miR-21 in hypertension mechanisms has been previously found to be apparent in RAAS, through the regulation of aldosterone secretion [34] and cardiac fibrosis, caused by ERK-MAP kinase activity [36] and PTEN/Smad targeting [35,37]. Therefore, it remains to be further clarified whether the specific mechanism through which miR-21 affects night blood pressure in our population applies to one of the aforementioned pathways. 

Chios mastic is a natural Greek product, with bioactive compounds [41] which exert anti-inflammatory [51,52], hypolipidemic [47,48], antiatherogenic [43], and antioxidant [53,54] activity. The resinous exudate, isolated from Chios mastic, is characterized by an extremely multifarious chemical composition consisting mainly of triterpenes, volatile compounds, and some phenolic compounds [53]. There is evidence suggesting the interaction of Chios mastic’s compounds with miR-21. Kubatka et al. investigated the chemotherapeutic effect of *Cinnamomum zeylanicum*, a natural product containing some of the monoterpenes found in mastic, such as linalool, limonene, and α-terpineol, on animal models with breast carcinoma. A low-dose of cinnamon (8.1 mg daily) resulted in miR-21 expression decrease [55]. The same team evaluated the onco-protective effect of *Salvia officinalis* in the same animal model. One of the constituents found in *S. officinalis* was oleanolic acid, a pentacyclic triterpene also isolated from the neutral fraction of Chios mastic resin. The high-dose of *S. officinalis* (1% *w*/*w*) significantly downregulated miR-21 in the rodent tumors [56]. Gallic acid is one of the phenolic compounds detected in Chios mastic [57]. Chung et al. showed that gallic acid inhibits intracellular miR-21 expression, thus leading to reduced VSMC proliferation and migration through the regulation of yjr PTEN/Akt signaling pathway [58]. In another study published by Hussein et al., miR-21 was examined as a key-regulator of fibrosis in the livers of rats, whereas gallic and ferulic acid were investigated for their protective effect. The administration of both phenolic compounds resulted in a downregulation of miR-21 via the interference with the TGF-β1/Smad3 pathway [59].

The current study was the first randomized clinical trial (RCT) examining the antihypertensive properties of a Chios mastic extract based on microRNA expression. Previous studies investigating Chios mastic’s effect on miR-21 expression have focused on inflammation-related conditions such as non-alcoholic fatty liver disease (MAST4HEALTH) [60] and inflammatory bowel disease (MASTIHA IBD-GR) [61]. Similar to our results, none of the aforementioned studies showed statistically significant difference in miR-21 levels when comparing Chios mastic and placebo groups. In our study, mean changes of miR-21 differed significantly between the placebo and the low-dose mastic group (*p* = 0.037) and the low-dose and high-dose group (*p* = 0.018) when adjusting for sex and age. Similar differences were observed when adjusting for age, sex, and BMI categories in the same group comparisons (*p* = 0.017 and *p* = 0.010, respectively). This finding could be due to the consideration of covariates, strongly related to hypertension onset and progress. A dose–response effect could explain the fraction of the patient population, with the intent to titrate the dose of mastic as long as the supplement is well-tolerated. Based on our measurements, we observed a decrease in miR-21 expression levels post-intervention in the low-dose mastic group and, interestingly, a slight non-significant increase in both the placebo and high-dose groups. This could be interpreted as dose–response evaluation since a specific dose of mastic seems to exhibit antihypertensive effect. Alternatively, we could assume that the small size of the study population could mask a clinically important effect on the high-dose group, but this needs further investigation via a follow-up research study. Both the three-month duration of our study and the quantity of 2800 mg of mastic supplementation are in accordance and within the spectrum of previous RCTs with mastic [42,52,60,62,63].

In our attempt to untangle the regulatory role of miR-21 in hypertension, we examined the possible correlations of miR-21 levels with hypertension indices and anthropometric characteristics. Linear regression analysis showed no statistically significant association of miR-21 levels at baseline for any of the measured anthropometric, clinical, and hemodynamic variables. However, statistically significant associations with nocturnal measurements were observed in the low-dose mastic group after the intervention. During night-time sleep, BP tends to decline by 10–20%, compared to daytime [64,65], a phenomenon known as dipping. Night-time hypertension, defined as an approximate BP greater than or equal to 120/70 mmHg, has been demonstrated by several studies as a more accurate prediction marker of cardiovascular risk, compared to 24 h and daytime BP [1,66], and is related to arterial stiffening [66]. Our results indicate a positive association of miR-21 levels with night-time SBP, also shown in the work of Cengiz et al. [22]. Intriguingly, night-time SBP has been previously associated with platelet-derived extracellular vesicles (pEVs) [67], which are known carriers of microRNAs [68], and they could possibly transfer miR-21. In our study, miR-21 levels were also positively associated with night-time PP and central SYSMAP, thus verifying the association of miR-21 with hypertension. These results contradict previous findings by Kontaraki et al., showing a negative correlation of miR-21 with 24 h DBP, 24 h mean PP, and mean BP [27]. PWV was negatively associated with miR-21 levels. Considering the induced expression levels of miR-21 in hypertensive individuals, it would have been expected to have a positive association with indices of hypertension. This contradiction might be attributed to endogenous repair mechanisms, not yet clarified. Surprisingly, Syed et al. [69] suggested the inclusion of miR-21 in the category of cardioprotective miRs, also called “protectomiRs” [70], based on the finding that miR-21 abrogation resulted in aldosterone/salt-mediated cardiac hypertrophy induction in miR21-knockout mice. However, this result was independent from hypertension in animal models and has not been thoroughly investigated in humans.

In the current study, we showed an upregulation of miR-21 in hypertensive individuals, verifying the prognostic value of this epigenetic biomarker in hypertension. Concerning the effect of Chios mastic on hypertension, through the implication of miR-21, we observed an association between miR-21 and night-time blood pressure measurements only when a low-dose of Chios mastic (1500 mg), adjunct to anti-hypertensive treatment, was administered. 

The aforementioned results should be interpreted considering several study limitations. In order to verify the findings of our study, a larger sample size is necessary so as to obtain a better representation of the population. Furthermore, considering that cardiovascular risk factors were well-controlled for all patients prior to intervention, pro-hypertensive mechanisms were practically hibernated, preventing the observation of Chios mastic’s additive effect. Moreover, microRNA assessment was conducted based on circulation levels and not on the affected tissues. However, these limitations could be counterpoised by the meticulously designed RCT, the high-specificity and reproducibility methodologies used for miR-21 extraction and quantification, and the perennial experience of the research group from previous clinical interventions.

## 4. Materials and Methods

### 4.1. Study Design

This research was conducted on a subset of volunteers with hypertension who participated in the HYPER-MASTIC study (https://www.hyper-mastic.eu, accessed on 17 November 2022), a cooperative scientific project of academic and corporate institutions. HYPER-MASTIC is a 3-month, randomized, double-blind, placebo-controlled clinical intervention, aiming to investigate Chios mastic’s antihypertensive action through the research, development, and commercial production of innovative phytotherapeutic supplements based on this Greek natural product. Recruitment and patient evaluation was performed by the Department of Clinical Therapeutics of the National and Kapodistrian University of Athens. Volunteer eligibility was determined based on the following characteristics: individuals of both sexes, aged 40–80 years, with stable or controlled arterial hypertension for at least two months prior to intervention. Hypertension was assessed based on 24 h monitoring measurements. Exclusion criteria included history of acute myocardial infarction, stroke or heart failure within the last 6 months prior to intervention, use of antioxidant-containing supplements, allergic reaction to Chios mastic, and history of autoimmune diseases, cancer, or an active infection.

Prior to their participation in the study, all individuals signed an informed consent form. Subsequently, they were randomly allocated into three intervention groups, which were formed as follows: low-dose mastic, high-dose mastic, and placebo. The low-dose group received two tablets on a daily basis, each containing 750 mg; the high-dose group received four tablets on a daily basis, each containing 700 mg; and the placebo group received either two or four placebo tablets daily. The dose of 2800 mg of Chios mastic was considered as an effective concentration for the observation of changes in arterial blood pressure based on recent results by Kontogiannis et al. [42]. All tablets were produced by IASIS Pharmaceuticals Hellas S.A. (https://iasispharma.com/, accessed on 17 November 2022). Blinding was maintained throughout the intervention on both participants, and scientific staff and randomization groups were revealed only after the intervention trial was complete.

The study was carried out according to the principles of the 18th World Medical Association (Helsinki, 1964) and the guidelines of the International Council for Harmonization, concerning Good Clinical Practice. The study protocol was reviewed and approved by the Harokopio University Ethics Committee (35-21/12/2021) and the Scientific Board of Alexandra Hospital (8/23-12-2021) and submitted on Clinical Trials.gov (Protocol ID: 251/12-05-2015).

Herein, data and plasma samples of fifty-seven (57) hypertensive patients that had completed the intervention up to the time of analysis were used. Forty-six (46) samples from normotensive individuals were derived from the HUA Biobank (located at Harokopio University) and were used as healthy controls.

### 4.2. Clinical, Anthropometric, and Lifestyle Assessment

Clinical characteristics and hemodynamic parameters of well-controlled patients with hypertension were assessed at baseline and after the 3-month intervention. Arterial stiffening of patients was assessed using the pulse wave velocity (PWV) measurement, a gold-standard method based on the calculation of the carotid and femoral pulse pressures and the time delay between the two measurements (Complior, Alam Medical, Saint-Quentin-Fallavier, France). For the analysis of PWV imaging results, the Brachial Analyzer for the Research Medical Imaging Applications Program (MIA-LLC, Coralville, IA, USA) was employed. Evaluation of 24 h arterial stiffening and arterial pressure fluctuation was conducted using a Mobil-O-graph monitor (Mobil-O-graph, IEM, Aachen, Germany), while the automated SphygmoCor System (AtCor Medical Pty Ltd., Sydney, Australia) was used for the assessment of aortic pressure waveforms and reflected waves. 

Medical history was recorded by experienced medical staff, while nutritional habits were documented through 24 h recalls and a food frequency questionnaire. Patients were instructed to maintain their usual diet and physical activity and document daily supplement consumption in order to monitor compliance to intervention. Anthropometric measurements included body weight (kg), height (cm), and body mass index (kg/m^2^) assessment. Physical activity levels were evaluated using the short version of the International Physical Activity Questionnaire (IPAQ) [71].

### 4.3. Blood Collection

Freshly isolated plasma samples collected in two distinct time-points—at baseline and upon intervention completion—were used. Blood was collected in tubes containing EDTA, which were then centrifuged for 10 min at 3000 rpm and 4 °C in order to separate plasma and serum specimens. Both plasma and serum were subsequently stored at −80 °C until further analysis. Plasma samples subjected to hemolysis were excluded from the analysis.

### 4.4. microRNA Quantification

Total RNA, including microRNAs, was extracted from 100 μL of plasma, using MagMAX™ mirVana™ Total RNA Isolation Kit (cat no A27828, Applied Biosystems™, ThermoFisher Scientific Inc., Waltham, MA, USA), according to the manufacturer’s protocol. This kit relies on magnetic bead technology, achieving reproducible high-quality RNA isolation. Implen P330 nanophotometer (Implen, GmbH, Munich, Germany) was used for the measurement of the extracted RNA’s optical density in order to calculate its concentration and reassure its purity. RNA samples with low concentration or purity were excluded from the analysis.

cDNA synthesis was performed using TaqMan™ Advanced miRNA cDNA Synthesis Kit (cat no A28007, Applied Biosystems™, ThermoFisher Scientific Inc., Waltham, MA, USA), according to the manufacturer’s protocol. The cDNA synthesis workflow consists of four stages of miRNA modifications—extension of the 3′ end via poly[A] addition, adaptor ligation in the 5′ end, reverse transcription using a universal primer, and finally, amplification—in order to increase the quantity of every reverse transcribed microRNA.

Quantification of microRNA-21 levels was performed using the quantitative real-time polymerase chain reaction (qRT-PCR) method. The reaction mix for each sample of amplified cDNAs included TaqMan^®^ Advanced miRNA Assays [for hsa-miR-21-5p (assay ID #477975_mir) for cel-miR-39-3p (assay ID #478293_mir)] and TaqMan^TM^ Fast Advanced Master Mix. Reactions were carried out in the StepOnePlus^TM^ System (ThermoFisher Scientific Inc.). Cycling conditions were as follows: 95 °C for 20 s, followed by 40 repeated cycles of 95 °C for 1 s and 60 °C for 20 s. Data analysis was performed using the ExpressionSuite™ Software (v1.3), a data-analysis tool which employs the comparative Ct (ΔΔCt) method for fast and precise assessment of relative gene expression. *Caenorhabditis elegans* microRNA-39 (cel-miR-39-3p) was added in every plasma sample during RNA extraction and was used as an exogenous control to ensure that miRNA quantification is not affected by the variability of samples. Exogenous normalization can efface several deviations of the experimental workflow and enhance the reliability of results. miR-21 levels of hypertensive and normotensive individuals were expressed as fold changes after comparison with a reference sample using the 2^−ΔΔCt^ method.

### 4.5. Statistical Analysis

Descriptive characteristics are presented at baseline for each intervention group. Categorical variables are presented as relative frequencies. Continuous variables were tested for normality using the Kolmogorov–Smirnov test and QQ plots. Variables following a normal distribution are presented as mean ± standard deviation, whereas those not following a normal distribution are presented as the median (interquartile range). Independent samples *t*-test and one-way ANOVA were employed for the comparison of differences among two and three groups, respectively. The covariates used for adjustment were sex and age. Paired samples *t*-test analysis was used in order to examine differences in miR-21 levels before and after the 3-month intervention for each group. Post hoc analysis was conducted using repeated measures ANOVA and the Bonferroni method (usually used for small sample sizes) in order to examine the differences between the intervention groups separately, as for the degree of changes in miR-21 levels. Linear and multilinear regression models (adjusted for age and sex) were used to examine possible association between miR-21 levels (independent variable) and anthropometric, clinical, hemodynamic, and vascular measurements (dependent variables). SPSS statistical software (version 27.0.1) was employed for statistical analysis, and the level of statistical significance was set at α = 0.05. Figures were designed using GraphPad Prism version 10.2.2 for Windows (GraphPad Software, Boston, MA USA, www.graphpad.com, accessed on 29 January 2023) and the package Plotly in R (Plotly Technologies Inc. Collaborative data science. Montréal, QC, Canada, 2015. https://plot.ly, accessed on 29 January 2023) [72].

## 5. Conclusions

The present research suggests a potential implication of miR-21 in the effect of Chios mastic on night-time blood pressure measurements. This intercross needs to be further elucidated in order to understand the underlying molecular pathways and to design targeted antihypertensive treatment strategies.

## Figures and Tables

**Table 1:** Baseline characteristics of the study population.

Baseline Characteristics	Placebo (N = 23)	Low-Dose of Mastic (N = 16)	High-Dose of Mastic (N = 18)	*p*
Age (years)	63.8 ± 6.372	58.38 ± 7.032	59.56 ± 9.288	0.065
Sex (m/f)	13/10	12/4	10/8	0.433
Smoking	30.4%	31.3%	27.8%	0.974
Years of smoking	9 (20)	5.5 (25)	3 (26)	0.982
DM	17.4%	18.8%	22.2%	0.929
Hyperlipidemia	69.6%	68.8%	61.1%	0.839
Years of HT	10 (24)	6 (8.9)	4 (15.3)	0.165
Weight (kg)	79 (14)	85 (20.5)	91 (19.7)	0.063
BMI (kg/m^2^)	26.7 (5.91)	30.3 (6.07)	30.04 (5.28)	0.114
miR-21 (pg/mL)	0.196 (0.3)	0.304 (0.4)	0.17 (0.3)	0.570
Mean 24-h SBP (mmHg)	122.2 ± 8.393	121.38 ± 7.429	125.22 ± 8.3	0.339
Mean 24-h DBP (mmHg)	76.39 ± 8.398	76.44 ± 8.181	78.44 ± 9.05	0.707
Daytime SBP (mmHg)	124.13 ± 8.374	123.63 ± 7.154	126.72 ± 8.608	0.478
Daytime DBP (mmHg)	78.22 ± 8.671	78.25 ± 8.226	80.22 ± 9.309	0.729
Daytime MAP (mmHg)	99.26 ± 7.852	99.13 ± 7.108	101.61 ± 8.396	0.562
Daytime HR (bpm)	71 (14)	69 (17)	71 (9)	0.676
Daytime PP (mmHg)	46 (10)	42.5 (10)	45.5 (8)	0.922
Daytime cSMAP (mmHg)	125.22 ± 8.613	124.69 ± 8.882	129 ± 8.561	0.274
Daytime cDMAP (mmHg)	79.78 ± 8.857	79.94 ± 7.759	82.28 ± 8.95	0.613
Daytime PWV (m/s)	8.96 ± 1.1	8.18 ± 0.886	8.63 ± 1.31	0.114
Night-time SBP (mmHg)	116.45 ± 11.677	115.06 ± 9.760	120.28 ± 12.136	0.376
Night-time DBP (mmHg)	70.68 ± 9.814	71.81 ± 9.752	72.61 ± 12.566	0.850
Night-time MAP (mmHg)	91.77 ± 9.875	91.56 ± 9.085	94.28 ± 11.871	0.682
Night-time HR (bpm)	62.27 ± 8.396	63.38 ± 13.089	64.11 ± 11.061	0.862
Night-time PP (mmHg)	44.5 (15)	41 (8)	46 (9)	0.258
Night-time cSMAP (mmHg)	125.32 ± 11.311	122 ± 8.315	127.94 ± 14.477	0.360
Night-time cDMAP (mmHg)	72.09 ± 9.481	74.67 ± 10.019	74.33 ± 12.649	0.720
Night-time PWV (m/s)	8.36 ± 2.18	7.91 ± 0.99	8.45 ± 1.33	0.605

The results are presented as mean ± SD for continuous variables with normal distribution, as median (IQR) for continuous variables not following a normal distribution, and as relative frequencies for categorical variables. m/f: males/females; DM: diabetes mellitus; HT: Hypertension; BMI: body mass index; SBP: systolic blood pressure; DBP: diastolic blood pressure; MAP: mean arterial pressure; HR: heart rate; PP: pulse pressure; cSMAP: central systolic MAP; PWV: pulse wave velocity. One-way ANOVA was used for the *p*-value calculation between mean levels of the presented variables in the three groups.

**Table 2:** Differences of miR-21 levels in the plasma of hypertensive patients at baseline and post-intervention.

Group	miR-21 Baseline (pg/mL)	miR-21 Post-Intervention (pg/mL)	*p*_time_ Baseline and Post-Intervention in Each Group	Group Comparisons	*p*_unadj_ between the Groups	*p*_adj1_ between the Groups	*p*_adj2_ between the Groups
Placebo	0.196 (0.3)	0.299 (0.3)	0.318	Placebo-Low dose	0.626	0.037 *	0.017 *
Low dose of mastic	0.304 (0.4)	0.268 (0.4)	0.287	Placebo-High-dose	1	0.872	0.343
High dose of mastic	0.172 (0.3)	0.211 (0.1)	0.869	Low dose-High-dose	0.399	0.018 *	0.010 *

The results are presented as median (IQR). *p*_time_: *p* from paired samples *t*-test for each intervention group between the miR-21 levels at baseline and post-intervention; *p*_unadj_; *p* from the comparison of miR-21 mean differences between groups, using repeated measures ANOVA (unadjusted) and post hoc Bonferroni for each association; *p*_adj1_; *p* from the comparison of miR-21 mean differences between groups, using repeated measures ANOVA (adjusted for age and sex) and post hoc Bonferroni for each association; *p*_adj2_; *p* from the comparison of miR-21 mean differences between groups, using repeated measures ANOVA (adjusted for age, sex, and ΒΜΙ categories) and post hoc Bonferroni for each association; *: *p*-value < 0.05

## Data Availability

The data that support the findings of this study are available upon request from the corresponding author G.V.D.

## Supplementary Materials

The following supporting information can be downloaded at https://www.mdpi.com/article/10.3390/ncrna10030033/s1: Table S1. Baseline clinical measurements of the study population; Table S2. Simple linear regression analysis of miR-21 levels, at baseline, with anthropometric characteristics and medical history of the study population; Table S3. Multilinear regression analysis of miR-21 levels with hemodynamic and vascular parameters of the study population, at baseline. The model is adjusted for sex and age; Table S4. Multilinear regression analysis of miR-21 levels, post-intervention, with hemodynamic and vascular parameters, for each intervention group. The model is adjusted for sex and age; Table S5. Multilinear regression of miR-21 levels, post-intervention, with clinical measurements for each group. The model is adjusted for sex and age; Table S6. Generalized linear models representing group-wise interactions with miR-21 levels, post-intervention; Table S7. Multilinear regression of miR-21 levels, post-intervention, with clinical measurements for each group.
