# Supplementary material for: Investigation of Antihypertensive Properties of Chios Mastic via Monitoring microRNA-21 Expression Levels in the Plasma of Well-Controlled Hypertensive Patients
Source: Noncoding RNA. 2024 May 31;10(3):33. doi: 10.3390/ncrna10030033 (PMC11207086; doi:10.3390/ncrna10030033)
Supplement: Supplementary file 1 [file ncrna-10-00033-s001.zip › ncrna-3006490-supplementary.pdf]

Supplementary data

Table S1. Baseline clinical measurements of the study population.

Clinical parameter	Placebo (N=23)	Low dose of mastic (N=16)	High dose of mastic (N=18)	p
Hematocrit (%)	43.2 (5)	44.55 (5)	42.35 (7)	0.288
Creatinine (mg/dL)	0.74 ± 0.2	0.82 ± 0.207	0.84 ± 0.221	0.302
LDH (U/L)	176 (46)	184.5 (36)	185 (74)	0.762
Total Cholesterol (mg/dL)	158 (29)	158 (41)	158.5 (83)	0.933
HDL(mg/dL)	56.17 ± 12.153	48.81 ± 11.28	50.56 ± 12.58	0.139
LDL (mg/dL)	78 (32)	82.5 (46)	85 (66)	0.917
ApoA1 (mg/dL)	152.13 ± 22.535	138.44 ± 20.013	140.78 ± 23.792	0.122
ApoB (mg/dL)	73 (24)	76.5 (24)	75.5 (32)	0.982
CRP (mg/dL)	1.28 (2)	1.15 (2)	0.91 (2)	0.524

The results are presented as mean ± SD for continuous variables with normal distribution and as median (IQR) for continuous variables not following a normal distribution. LDH: Lactate dehydrogenase; HDL: High density lipoprotein; LDL: Low density lipoprotein; ApoA1: Apolipoprotein A1; ApoB: Apolipoprotein B; CRP: C-reactive protein

Table S2. Simple linear regression analysis of miR-21 levels, at baseline, with anthropometric characteristics and medical history of the study population.

Parameter	Beta	Standard error	p
Age	-0.011	0.006	0.082
Sex	0.045	0.105	0.671
Smoking	-0.055	0.112	0.625
Years of smoking	0.002	0.003	0.531
DM	-0.075	0.064	0.247
Years of HT	0.004	0.005	0.406
Hyperlipidemia	-0.071	0.108	0.516

Table S3. Multilinear regression analysis of miR-21 levels with hemodynamic and vascular parameters of the study population, at baseline. The model is adjusted for sex and age.

Hemodynamic and vascular parameters	Beta	Standard error	p
Mean 24-hour SBP	-3.736	2.878	0.2
Mean 24-hour DBP	-1.645	2.821	0.562
Mean 24-hour MAP	-2.632	2.627	0.321
Mean 24-hour HR	-5.555	3.602	0.129
Mean 24-hour PP	-2.06	2.234	0.361

Hemodynamic and vascular parameters	Beta	Standard error	p
Mean 24-hour central systolic MAP	-3.389	6.268	0.591
Mean 24-hour central diastolic MAP	-1.452	2.765	0.602
Mean 24-hour PWV	-0.178	0.147	0.231
Day-time SBP	-3.382	2.850	0.241
Day-time DBP	-1.594	2.855	0.579
Day-time MAP	-2.394	2.617	0.364
Day-time HR	-6.358	3.662	0.088
Day-time PP	-1.551	2.239	0.492
Day-time central systolic MAP	-1.369	3.165	0.667
Day-time central diastolic MAP	-1.519	2.741	0.582
Day-time PWV	-0.184	0.143	0.203
Night-time SBP	-4.990	4.119	0.231
Night-time DBP	-1.410	3.739	0.708
Night-time MAP	-3.008	3.685	0.418
Night-time HR	-4.516	3.644	0.221
Night-time PP	-3.796	2.647	0.158
Night-time central systolic MAP	-4.275	4.294	0.324
Night-time central diastolic MAP	-0.806	3.664	0.827
Night-time PWV	0.010	0.459	0.982
Systole dipping	0.639	2.543	0.803
Diastole dipping	-0.364	3.387	0.915

SBP: Systolic blood pressure; DBP: Diastolic blood pressure; MAP: Mean arterial pressure; HR: Heart rate; PP: Pulse pressure; PWV: Pulse wave velocity

Table S4. Multilinear regression analysis of miR-21 levels, post-intervention, with hemodynamic and vascular parameters, for each intervention group. The model is adjusted for sex and age.

Placebo			
**Hemodynamic and vascular parameters**	**Beta**	**Standard error**	**p**
Mean 24-hour SBP	1.390	7.990	0.864
Mean 24-hour DBP	6.012	6.967	0.4
Mean 24-hour MAP	4.022	6.931	0.569
Mean 24-hour HR	3.710	7.799	0.640
Mean 24-hour PP	-4.514	5.850	0.450
Mean 24-hour central systolic MAP	-0.762	7.520	0.920
Mean 24-hour central diastolic MAP	7.038	6.818	0.316
Mean 24-hour PWV	0.081	0.242	0.742
Day-time SBP	-0.264	8.667	0.976
Day-time DBP	4.321	7.927	0.592

Day-time MAP	2.282	7.603	0.767
Day-time HR	1.077	8.268	0.898
Day-time PP	-4.432	6.908	0.529
Day-time central systolic MAP	-0.250	8.002	0.975
Day-time central diastolic MAP	4.386	8.445	0.610
Day-time PWV	0.079	0.277	0.779
Night-time SBP	7.505	10.706	0.492
Night-time DBP	12.428	7.927	0.134
Night-time MAP	10.054	9.074	0.282
Night-time HR	12.900	8.089	0.128
Night-time PP	-5.644	5.179	0.290
Night-time central systolic MAP	0.532	10.990	0.962
Night-time central diastolic MAP	13.482	7.612	0.093
Night-time PWV	0.162	0.334	0.633
Systole dipping	-5.902	8.335	0.488
Diastole dipping	-10.682	9.645	0.283

**Low dose of mastic**			
**Hemodynamic and vascular parameters**	**Beta**	**Standard error**	**p**
Mean 24-hour SBP	0.662	2.288	0.778
Mean 24-hour DBP	-0.641	2.205	0.777
Mean 24-hour MAP	0.161	1.976	0.937
Mean 24-hour HR	-1.716	2.145	0.441
Mean 24-hour PP	1.281	2.180	0.569
Mean 24-hour central systolic MAP	1.225	2.501	0.634
Mean 24-hour central diastolic MAP	-1.632	2.186	0.471
Mean 24-hour PWV	0.002	0.092	0.986
Day-time SBP	-0.066	2.178	0.976
Day-time DBP	-1.181	2.191	0.601
Day-time MAP	-0.508	1.942	0.798
Day-time HR	-2.323	2.146	0.302
Day-time PP	1.087	2.048	0.606
Day-time central systolic MAP	0.821	2.541	0.753
Day-time central diastolic MAP	-2.164	2.020	0.307
Day-time PWV	-0.033	0.083	0.702
Night-time SBP	17.870	7.175	**0.032**
Night-time DBP	-3.503	8.010	0.671
Night-time MAP	6.412	6.871	0.373
Night-time HR	-1.813	7.666	0.818
Night-time PP	20.968	6.014	**0.006**
Night-time central systolic MAP	17.706	6.472	**0.023**
Night-time central diastolic MAP	-3.071	8.885	0.738
Night-time PWV	-1.783	0.258	**<0.001**
Systole dipping	-7.776	3.987	0.080
Diastole dipping	-6.706	5.090	0.217

High dose of mastic			
**Hemodynamic and vascular parameters**	**Beta**	**Standard error**	**p**
Mean 24-hour SBP	-20.838	11.284	0.095
Mean 24-hour DBP	-13.039	11.455	0.282
Mean 24-hour MAP	-17.421	8.274	0.061
Mean 24-hour HR	8.291	17.698	0.649
Mean 24-hour PP	-7.610	15.786	0.640
Mean 24-hour central systolic MAP	-29.101	20.426	0.185
Mean 24-hour central diastolic MAP	-11.314	11.806	0.360
Mean 24-hour PWV	-1.533	3.104	0.631
Day-time SBP	-22.394	14.416	0.151
Day-time DBP	-16.151	10.994	0.686
Day-time MAP	-1.996	9.558	0.063
Day-time HR	10.535	16.545	0.562
Day-time PP	-5.803	16.562	0.733
Day-time central systolic MAP	-31.153	20.565	0.161
Day-time central diastolic MAP	-13.712	11.809	0.273
Day-time PWV	-1.650	3.050	0.599
Night-time SBP	-17.752	12.032	0.171
Night-time DBP	-4.077	18.597	0.831
Night-time MAP	-9.657	13.935	0.504
Night-time HR	4.108	20.012	0.841
Night-time PP	-14.073	15.471	0.384
Night-time central systolic MAP	-24.431	24.983	0.351
Night-time central diastolic MAP	-5.463	17.950	0.767
Night-time PWV	-1.397	3.275	0.678
Systole dipping	-3.551	13.274	0.794
Diastole dipping	-12.624	18.128	0.501

Table S5. Multilinear regression of miR-21 levels, post-transcription, with clinical measurements for each group. The model is adjusted for sex and age.

Placebo			
**Clinical measurements**	**Beta**	**Standard error**	**p**
Hematocrit	-4.112	2.635	0.136
Creatinine	-0.098	0.190	0.613
LDH	-10.00	49.435	0.842
Total Cholesterol	47.140	47.186	0.331
HDL	-8.284	14.183	0.566
LDL	51.266	34.138	0.150
ApoA1	-5.115	33.952	0.882
ApoB	43.978	27.861	0.132
CRP	2.682	5.757	0.647
**Low dose of mastic**			

Clinical measurements	Beta	Standard error	p
Hematocrit	0.022	0.706	0.976
Creatinine	-0.024	0.036	0.527
LDH	9.722	7.681	0.232
Total Cholesterol	-14.638	8.568	0.116
HDL	-1.184	3.737	0.757
LDL	-13.704	7.883	0.110
ApoA1	0.420	5.907	0.945
ApoB	-6.736	4.233	0.140
CRP	-1.553	5.604	0.787
**High dose of mastic**			
**Clinical measurements**	**Beta**	**Standard error**	**p**
Hematocrit	-4.166	4.580	0.383
Creatinine	-0.337	0.310	0,301
LDH	84.098	59.990	0.189
Total Cholesterol	81.534	46.738	0.109
HDL	-7.404	20.143	0.720
LDL	60.798	45.384	0.207
ApoA1	6.636	38.207	0.865
ApoB	28.841	25.114	0.275
CRP	1.202	3.525	0.740

Table S6. Generalized linear models representing group-wise interactions with miR-21 levels, post-intervention. Each row represents a different model, examining the prediction of hemodynamic and vascular parameters by using the interaction of each group with miR-21 levels as independent variables and as confounding factors age and sex.

Hemodynamic and vascular parameters	Group	Beta	Standard error	p
Mean 24-hour SBP	Placebo	0.119	6.884	0.986
	Low-dose	0.895	2.067	0.665
	High-dose	-21.283	11.58	0.066
Mean 24-hour DBP	Placebo	5.53	6.23	0.375
	Low-dose	-0.133	1.871	0.943
	High-dose	-13.082	10.48	0.212
Mean 24-hour MAP	Placebo	3.277	5.6543	0.562
	Low-dose	0.548	1.698	0.747
	High-dose	-17.669	9.511	0.063
Mean 24-hour HR	Placebo	-0.249	7.988	0.975
	Low-dose	-1.337	2.399	0.577
	High-dose	8.979	13.437	0.504
Mean 24-hour PP	Placebo	-5.324	6.724	0.428
	Low-dose	1.020	2.019	0.613
	High-dose	-8.165	11.31	0.470
Mean 24-hour central systolic MAP	Placebo	-0.641	8.212	0.923
	Low-dose	1.123	2.466	0.649

Hemodynamic and vascular parameters	Group	Beta	Standard error	p
	High-dose	-30.085	13.813	**0.029**
Mean 24-hour central diastolic MAP	Placebo	6.517	6.194	0.293
	Low-dose	-1.165	1.860	0.531
	High-dose	-10.962	10.419	0.293
Mean 24-hour PWV	Placebo	-0.042	0.331	0.9
	Low-dose	-0.017	0.099	0.867
	High-dose	-0.178	0.557	0.750
Day-time SBP	Placebo	-5.965	6.478	0.357
	Low-dose	-1.257	1.947	0.518
	High-dose	2.247	10.905	0.837
Day-time DBP	Placebo	3.197	6.348	0.615
	Low-dose	-1.677	1.908	0.379
	High-dose	2.319	10.686	0.828
Day-time MAP	Placebo	-0.839	5.824	0.885
	Low-dose	-1.467	1.751	0.402
	High-dose	1.386	9.805	0.888
Day-time HR	Placebo	1.227	9.115	0.893
	Low-dose	-1.001	2.739	0.714
	High-dose	2.658	15.345	0.862
Day-time PP	Placebo	-9.904	5.387	0.066
	Low-dose	0.590	1.619	0.715
	High-dose	1.368	9.069	0.880
Day-time central systolic MAP	Placebo	-9.286	7.395	0.209
	Low-dose	-0.242	2.222	0.913
	High-dose	-2.697	12.44	0.828
Day-time central diastolic MAP	Placebo	2.00	6.103	0.743
	Low-dose	-1.906	1.834	0.299
	High-dose	1.614	10.274	0.875
Day-time PWV	Placebo	0.127	0.957	0.895
	Low-dose	-0.012	0.287	0.965
	High-dose	-2.196	1.611	0.173
Night-time SBP	Placebo	4.3	8.589	0.617
	Low-dose	17.466	8.2001	**0.033**
	High-dose	-16.096	14.447	0.265
Night-time DBP	Placebo	10.061	8.065	0.212
	Low-dose	-3.660	7.001	0.635
	High-dose	-2.537	13.566	0.852
Night-time MAP	Placebo	7.264	7.763	0.349
	Low-dose	6.150	7.412	0.407
	High-dose	-7.955	13.058	0.542
Night-time HR	Placebo	8.361	8.834	0.344
	Low-dose	-1.220	8.434	0.885

Hemodynamic and vascular parameters	Group	Beta	Standard error	p
	High-dose	5.553	14.859	0.709
Night-time PP	Placebo	-6.515	6.030	0.280
	Low-dose	20.766	5.757	**<0.001**
	High-dose	-13.877	10.143	0.171
Night-time central systolic MAP	Placebo	-1.179	10.255	0.908
	Low-dose	17.253	9.87	**0.08**
	High-dose	-24.833	17.246	0.150
Night-time central diastolic MAP	Placebo	11.299	7.999	0.158
	Low-dose	-3.445	7.699	0.655
	High-dose	-4.497	13.453	0.738
Night-time PWV	Placebo	0.113	1.065	0.915
	Low-dose	-1.778	0.320	**<0.001**
	High-dose	-1.855	1.793	0.301
Systole dipping	Placebo	-2.935	6.956	0.673
	Low-dose	-7.702	6.647	0.247
	High-dose	-6.036	11.711	0.606
Diastole dipping	Placebo	-6.248	8.963	0.486
	Low-dose	-6.338	8.565	0.459
	High-dose	-15.517	15.090	0.304

Table S7. Multilinear regression of miR-21 levels, post-intervention, with clinical measurements for each group. Each row represents a different regression model, examining the prediction of miR-21 levels using each time as a dependent variable one of the parameters and the confounding factors age and sex.

Clinical measurements	Group	Beta	Standard error	p
Hematocrit	placebo	-3.292	2.393	0.169
	low dose	0.048	0.719	0.946
	high dose	-4.317	4.028	0.284
Creatinine	placebo	-0.093	0.152	0.543
	low dose	-0.022	0.46	0.639
	high dose	0.371	0.257	0.150
LDH	placebo	-14.008	35.807	0.696
	low dose	10.569	10.761	0.326
	high dose	82.530	60.281	0.171
Total Cholesterol	placebo	44.560	33.094	0.178
	low dose	-14.560	9.946	0.144
	high dose	82.697	55.713	0.138
HDL	placebo	-8.284	11.457	0.441
	low dose	-0.652	3.443	0.850
	high dose	-7.683	19.288	0.690
LDL	placebo	50.627	27.058	0.061
	low dose	-13.818	8.132	0.089

**Clinical measurements**	**Group**	**Beta**	**Standard error**	**p**
	high dose	63.905	45.552	0.161
ApoA1	placebo	-7.145	24.118	0.767
	low dose	1.347	7.248	0.853
	high dose	8.132	40.602	0.841
ApoB	placebo	44.696	18.733	0.132
	low dose	-7.013	5.630	**0.017**
	high dose	27.569	31.538	0.213
CRP	placebo	4.089	9.732	0.674
	low dose	-2.063	2.924	0.481
	high dose	3.498	16.384	0.831
